# A One Health Framework for Proteomics Across the Tree of Life to Advance Food Security, Animal Health, and Ecosystem Resilience

**DOI:** 10.3390/proteomes14030032

**Published:** 2026-06-24

**Authors:** Tarun Mishra, Ritudhwaj Tiwari, Tuyelee Das, Maneesh Lingwan

**Affiliations:** 1 Department of Microbiology and Immunology, Carver College of Medicine, University of Iowa, Iowa City, IA 52242, USA; 2 Division of Infectious Diseases, Department of Medicine, Washington University School of Medicine, Saint Louis, MO 63110, USA; 3 University Centre for Research and Development, Chandigarh University, Mohali 140413, Punjab, India; tuyeleedas25@gmail.com; 4 Donald Danforth Plant Science Center, Saint Louis, MO 63132, USA

**Keywords:** one health proteomics, proteogenomic, mass spectrometry, animal health, crop biofortification, metaproteomics

## Abstract

As global ecosystems and food systems face unprecedented anthropogenic and climatic challenges, there is a demand for an integrated understanding of biological systems. Proteomics has emerged as a definitive approach offering a direct view of the molecular phenotype, yet it is traditionally separated into plant and animal disciplines. With recent advances in mass spectrometry (MS) and bioinformatics tools, this prospective review proposes that combining a One Health proteomics approach with deep-learning data analysis can revolutionize global food security, animal productivity, and ecosystem health by uncovering proteoform signatures that drive resilience across life. The potential of a unified One Health proteomic framework, highlighting major developments, including 4D proteomics, Data-Independent Acquisition (DIA), and single-cell resolution, and emphasizes their capacity to resolve the complex proteoform landscape across kingdoms. Review emphasizes the applications of proteogenomics as a cross-disciplinary tool to improve genome annotations, explain evolutionary differences, discover biomarkers in animals and resolve complex signaling networks in plants under stress. Nevertheless, contemporary proteogenomics methods still show limitations in their ability to comprehensively resolve proteoforms due to the fact that the use of peptide-based approaches makes it difficult to fully appreciate the post-translational modifications specific to each protein isoform. We show that One Health proteomics will provide a transformative roadmap for deciphering the functional proteoform signatures that underpin resilience across the tree of life.

## 1. Introduction

The completion of genome sequencing projects opened many new opportunities to decode biological systems; however, it quickly became evident that genomic information alone cannot fully explain phenotype. Protein abundance, post-translational modifications (PTMs), localization, turnover, and interaction networks ultimately determine functional outcomes. The discrepancy between transcript and protein levels, combined with the complexity introduced by PTMs, highlights the need for direct protein measurement [1,2]. Mass spectrometry (MS)-based proteomics has appeared as the principal technology for bridging this genotype-to-phenotype gap. Improvements in acquisition approaches from stochastic data-dependent acquisition (DDA) [3] to highly reproducible data-independent acquisition (DIA) workflows have enhanced proteome depth and quantitative reproducibility [4]. The integration of trapped ion mobility as a fourth, orthogonal separation dimension enables four-dimensional (4D) proteomics [5], thereby enhancing the separation of co-eluting peptides and increasing sensitivity for low-abundance peptides in complex samples [6,7]. These technical innovations, when combined with sensitive PTM enrichment strategies [8], allow proteomics to capture regulatory processes that directly modify phenotype across biological systems. Rising spatial and single-cell proteomics approaches prolong this ability to cellular heterogeneity, allowing for the dissection of tissue microenvironments and rare cell populations [9,10]. Together, these advancements position proteomics as an important component that combines genomic potential with apparent biological traits. This review defines One Health proteomics as the integrated use of proteomic analyses to connect molecular phenotypes across humans, animals, plants, microbes, and their shared environments. The focus is intentionally forward-looking, and is not aimed at cataloging every plant or animal proteomics study exhaustively. Instead, it examines how MS-based workflows can be structured within a flexible framework that supports three interconnected goals of food security, animal health, and ecosystem resilience. This approach necessitates explicitly including microbiomes and environmental samples, as many One Health risks, such as zoonotic spillover, antimicrobial resistance, soil nutrient cycling, water quality, and climate stress, operate through host–microbe–environment interfaces rather than through individual organisms.

**Figure 1:**
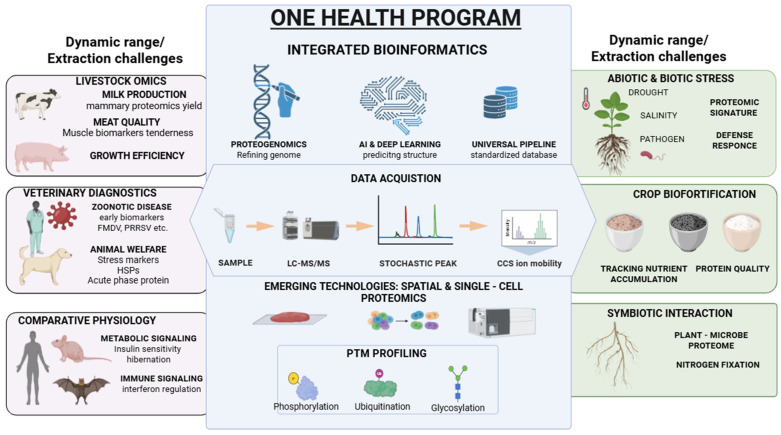
One Health proteomics framework linking plant, animal, microbial, and environmental compartments. The framework begins with connected biological samples, applies harmonized MS-based workflows and findable, accessible, interoperable, and reusable (FAIR) data practices, and translates conserved or system-specific protein signatures into outputs relevant to food security, animal health, environmental surveillance, and ecosystem resilience [14,25,26]. Illustration created using BioRender.

The One Health approach highlights how human, animal, and environmental health are all connected within shared ecosystems. In this context, studying both plant and animal proteomics together is not just interesting but important (Figure 1). Many global challenges, such as food security, zoonotic diseases, antimicrobial resistance, and climate change, are linked through molecular processes controlled by proteins. Proteins regulate metabolism, immune defense, and stress responses [11]. In animal systems, mass spectrometry-based proteomics improves animal productivity. It also helps with disease resistance and veterinary diagnostics in animal health and wildlife [12,13]. Comparative physiology employs animal models to elucidate conserved metabolic and immune signaling pathways that are relevant to both humans and animals [14]. In plants, proteomic studies have identified signatures of drought, salinity, ß and pathogen defense responses, which are critical for crop robustness [15,16]. MS-based methods also support crop biofortification by monitoring nutrient accumulation and seed protein composition [17]. They also point to plant–microbe partnerships, such as nitrogen fixation. This helps maintain sustainable agriculture [18,19]. Despite differences in organismal complexity and tissue structure, plant and animal proteomics face similar analytical challenges. The extreme range of protein amount and extraction barriers is the main issue [20]. In animals, highly abundant plasma proteins, and in plants, major storage or photosynthetic proteins [21] hide low-abundance regulatory proteins. Tackling these problems with enrichment strategies and improved sample preparation workflows allows for fairer comparisons between kingdoms. Therefore, MS-based proteomics provides a shared technological platform for advancing One Health goals.

Historically, plant science and animal research have been considered to be separate areas of study. Mostly, agricultural studies focus on crop productivity, while animal studies focus on developing treatments for animal diseases. However, current MS technologies and computational methods have developed new ways to connect these areas of research. The integration of DIA, ion mobility-enhanced 4D proteomics, and advanced bioinformatics is blurring taxonomic boundaries [22]. Proteogenomics facilitates this by using MS-derived peptides to improve, validate, and update genome annotations for plants and animals [23]. Concurrently, artificial intelligence and deep learning tools, including structure prediction tools such as AlphaFold [24], are improving functional annotation and helping structure-based proteomic analyses across taxa. The merging of previously distinct research domains is also supported by efforts to develop interoperable databases and standardized analytical pipelines. As both animal and plant research frequently use MS workflows, proteomics is emerging as a common technique that connects agricultural research, ecosystem resilience, and biomedical innovation. In this review, we explore how technological frontiers in MS, from DDA to DIA and 4D-proteomics, combine with spatial and single-cell analysis, PTM profiling, and cross-kingdom comparative challenges (Figure 1). In spite of such advancements, there remains one significant drawback: the vast majority of MS-based and proteogenomics techniques have yet to be able to determine complete proteoforms.

A practical One Health proteomics framework can be organized into five linked pillars. First, studies should begin with a shared cross-sector question, such as how heat stress affects crop productivity, livestock welfare, pathogen pressure, and soil or aquatic microbiome function. Second, sampling should capture connected compartments rather than isolated organisms—for example, host tissues, biofluids, rhizosphere or gut microbiomes, feed or plant material, and relevant environmental matrices. Third, proteomic workflows should be harmonized through standardized extraction, digestion, acquisition, quality control, and metadata reporting so that differences reflect biology rather than laboratory practice. Fourth, interpretation should focus on comparable functional modules across kingdoms, including stress signaling, immune or defense activation, energy metabolism, nutrient transport, xenobiotic response, and proteostasis. Fifth, outputs should be translated into actionable indicators such as biomarker panels, pathogen or antimicrobial-resistance surveillance signatures, welfare indicators, crop resilience markers, or ecosystem-monitoring metrics. This structure converts One Health from a broad concept into an operational proteomics pipeline.

## 2. Technical Frontiers in Mass Spectrometry

### 2.1. Reforming the Path from DDA to DIA and 4D-Proteomics

Over the past few years, MS-based proteomics has seen transformative advancements that have enhanced proteome coverage, quantification accuracy and broadened biological insights. The major point of progress has been the transition in acquisition strategies from DDA to DIA, along with the incorporation of IMS, collectively enabling the emergence of 4D proteomics. These developments collectively enhance analytical depth and reproducibility, enabling the study of complex biological systems with exceptional resolution [27]. DDA has long been central to discovery proteomics. It selects the most intense peptide ions for MS/MS, but faces limitations like stochastic precursor choice, missing low-abundance peptides, and a limited dynamic range where abundant peptides dominate [28,29]. DIA has emerged as a powerful alternative tool to overcome these limitations. In DIA, the instrument breaks ions across set *m*/*z* ranges rather than selecting individual precursor ions. This method collects broad, unbiased fragmentation data across the whole detectable mass range [30]. As a result, this approach substantially improves quantitative accuracy and runs-to-run reproducibility, enabling deeper and more reliable proteome coverage across large cohorts of samples [31]. Although DIA produces highly multiplexed spectra, advances in computational analysis, spectral libraries, and machine learning now enable us to identify and measure proteins quickly and accurately, surpassing DDA workflows [32].

### 2.2. Ion Mobility and the Advent of 4D-Proteomics

A parallel frontier in mass spectrometry is the incorporation of ion mobility separation (IMS) into the analytical workflow. Ion mobility adds an orthogonal dimension of separation based on the gas-phase mobility of ions, effectively differentiating ions by size, shape, and charge in milliseconds before mass analysis [33]. When combined with liquid chromatography and high-resolution MS, ion mobility expands proteomic analysis into four dimensions: retention time (LC), mass-to-charge ratio (*m*/*z*), fragment ion spectra (MS/MS), and ion mobility (drift time). This 4D proteomics improves analytical specificity by improving precursor and fragment separation, reducing spectral congestion, which is especially beneficial in DIA, where co-fragmentation is inherent. Collision cross-sections act as a unique, three-dimensional “fingerprint” for molecules, improving the accuracy of peptide identification and database-building. Faster, more efficient separations are achieved with IMS on the millisecond timescale, making it compatible with high-throughput methods [34].

Technologies such as trapped ion mobility spectrometry (TIMS) and structures for lossless ion manipulation (SLIM) have pushed the performance envelope, yielding narrower ion mobility peaks and higher resolving power. Notably, parallel accumulation–serial fragmentation (PASEF) leverages TIMS to further increase sequencing speed and sensitivity by synchronizing ion release with MS/MS acquisition [35,36]. Combining DIA with ion mobility separation is a game-changer in MS. DIA enhances depth by sampling all precursors, lowering complexity, and increasing specificity. Together, these techs boost robustness, coverage, and reproducibility while reducing time. Advances like variable-window DIA, ion mobility optimization, machine learning for spectral deconvolution, and better tools keep the field progressing.

### 2.3. Mapping the Cellular Landscape at Spatial Resolution

Proteomic mapping with cellular and subcellular resolution is central to decoding tissue organization, cellular heterogeneity, and disease. Conventional bulk proteomics mixes thousands to millions of cells, generates averaged signals, loses spatial information, and has masking effects on rare cell populations. Spatial proteomics has emerged in response to this limitation by maintaining tissue architecture while allowing for protein identification and quantification directly within designated anatomical regions or microenvironments [37]. Approaches such as imaging mass spectrometry techniques (matrix-assisted laser desorption/ionization and secondary ion mass spectrometry) enable the label-free mapping of proteins, peptides, and metabolites across tissue sections that reveal spatial gradients and molecular niches associated with development, cancer, and immune responses [38]. Antibody-derived complementary approaches, like imaging mass cytometry and multiplexed ion beam imaging, rely on metal-tagged antibodies and mass spectrometry-based detection to quantify dozens of proteins in tandem with subcellular resolution [39,40]. These targeted spatial proteomics platforms offer potential for the high-dimensional phenotyping of tissues with spatial fidelity intact and are thus powerful in characterizing tumor microenvironments and the organization of immune cells. Meanwhile, advances in laser capture microdissection (LCM) combined with ultrasensitive LC–MS workflows have made the deep, untargeted proteomic profiling of spatially resolved regions of tissues possible, bridging the gap between discovery proteomics and histopathology [41].

In parallel, single-cell proteomics has rapidly evolved to directly study protein abundance in individual cells, overcoming challenges associated with minute sample amounts and extreme dynamic range. Innovations in sample preparation, nano LC, ultra-low-flow electrospray ionization, and high-speed, high-sensitivity mass spectrometers have made it possible to detect hundreds to thousands of proteins per cell [42,43]. Isobaric labeling strategies like SCoPE-MS and its derivatives use carrier channels to improve peptide detectability while retaining single-cell quantitative information, whereas emerging label-free approaches aim to minimize ratio compression and improve absolute quantification [44]. The combination of spatial and single-cell proteomics offers a unique perspective of how proteins are organized at different levels. The use of these technologies allows us to understand cellular diversity, how cells within tissues relate to each other, and the biological processes as they continue to change. Both are fundamental to the development of systems biology and precision medicine.

### 2.4. Protein Functions and Post-Translational Modifications (PTMs) Profiling

PTMs greatly expand proteomic complexity by regulating protein activity, stability, localization, and interactions. Phosphorylation, ubiquitination, and glycosylation are among the most studied PTMs, playing central roles in cellular signaling, proteostasis, and intercellular communication across all domains of life. PTMs are extensively analyzed using MS. MS offers sensitivity, selectivity, and localization to identify PTM sites; however, due to the variety of chemical modifications and their low abundance, innovative enrichment methods and advanced computational tools are needed for a comprehensive, quantitative PTM analysis [30].

Phosphorylation is the most explored PTM required for signal transduction in eukaryotic organisms, mainly in animal and plant organisms, through a complex regulatory network of kinases and phosphatases, which control development and the physiological response of the organism to environmental stress. Eukaryotes mainly include phosphorylate serine, threonine, and tyrosine. In contrast, prokaryotes and archaea target histidine and aspartate in two-component pathways, posing challenges for analysis due to acid lability [45]. Identifying sites of phosphorylation in phosphopeptides using advanced techniques (e.g., IMAC, TiO_2_) coupled with high-resolution mass spectrometry and site-localization algorithms has enabled researchers to provide extensive and deep coverage of the phosphoproteome and confirm the presence of both conserved signaling motifs, as well as regulatory architectures that are unique to specific kingdoms [26,46]. Ubiquitination and ubiquitin-like modifications add an additional layer of regulatory complexity by modulating signaling processes through protein turnover and trafficking. Through an enzyme cascade, proteins become ubiquitinylated (or Ubl-modified) via the addition of ubiquitin (or Ubl) to target substrates in a hierarchical fashion with varying patterns of poly-ubiquitin chain establishment, which can fulfil unique biological functions. Large-scale ubiquitinome profiling typically relies on the antibody-based enrichment of di-glycine-modified lysine residues following tryptic digestion, enabling precise site identification [47]. In comparison to eukaryotes, prokaryotes lack canonical ubiquitin; however, they do have analogous bacterial pupylation. Although pupylation and ubiquitin have similar functions in protein quality control, they differ significantly in the enzymology and chemistry of the modification process [48,49]. Comparative PTM proteomic studies indicate that functional similarities exist in protein degradation pathways across the three domains (eubacteria, archaea, and eukaryotes).

Glycosylation is one of the most structurally diverse and analytically challenging PTMs, which regulates protein folding, stability, cell–cell recognition, and host–pathogen interactions [50,51]. Eukaryotic glycosylation, particularly N- and O-linked forms, is highly heterogeneous and differs between tissues. Glycopeptides need to be enriched and analyzed using specialized fragmentation methods, like high-energy collisional dissociation (HCD), electron-transfer dissociation (ETD), and ETD with supplemental HCD (EThcD) for confident site assignment [52]. While glycosylation was thought to only occur in eukaryotes, a large variety also occurs in bacteria and archaea, but with simpler or altered glycan structures, reflecting evolutionary adaptation [53]. Cross-kingdom glycoproteomics has provided insights into the evolution of glycosylation machinery and its role in environmental adaptation, immune evasion, and pathogenicity. These studies highlight conserved regulatory principles and kingdom-specific innovations, highlighting the importance of PTM profiling to understand cellular regulation, evolutionary biology, and disease.

### 2.5. Proteoform–Proteogenomics Interface

Proteoforms, the specific molecular forms of proteins resulting from splicing, genetic variation, truncation, and numerous post-translational modifications, increasingly prove to be the actual functional units of the proteome, extending well beyond the functional implications of the underlying genes and peptides [54]. Current ‘bottom-up’ proteomics approaches, which use digested peptides to predict proteins, cannot accurately link peptide data to specific proteoforms, which limits our understanding of isoform switching, combinatorial PTM events, and sequence variation in phenotypes and diseases [55]. This “proteoform challenge” has stimulated increasing interest in top-down proteomics (TDP), in which intact proteins are analyzed directly using high-resolution mass spectrometry [56]. Recent advances in instrumentation, multidimensional intact protein separations, fragmentation strategies, and computational tools have significantly improved the depth and robustness of TDP, enabling the characterization of thousands of proteoforms across plasma, cell lines, tissues, and even single cells. For example, deep TDP of isogenic colorectal cancer cell lines has identified over 23,000 proteoforms, revealing proteoform-level differences associated with metastatic potential [57]. Similarly, nanoparticle-based enrichment strategies have enhanced the detection of low-abundance plasma proteoforms across wide dynamic ranges. Despite these advances, TDP also faces substantial technical and analytical challenges. These include difficulties in solubilizing and separating large or membrane proteins, the extreme dynamic range of proteoforms in complex biofluids, spectral congestion from highly modified or closely related proteoforms, and the lack of high-throughput, standardized data analysis pipelines [58,59]. Conceptual and methodological development continues to highlight integrative approaches involving both bottom-up and top-down measurements, those consistent with our current understanding of proteome complexity and those prioritizing analytical rigor over speed to achieve truly proteoform-resolved maps for both basic and translational research.

## 3. Bottleneck of Precision and Comparative Challenges in Sample Preparation

In mass spectrometry-based proteomics, sample preparation is a crucial step that has a bigger impact on proteome coverage and data quality than the mass spectrometer itself. Efficient protein extraction, a reduction in sample complexity, the preservation of biologically significant modifications, and compatibility with enzymatic digestion and MS ionization are all necessary for effective sample preparation. Due to variations in tissue composition, cellular architecture, and protein abundance distributions, these requirements differ significantly between biological kingdoms. Therefore, kingdom-specific but conceptually harmonized workflows are required for comparative proteomics between plants and animals to guarantee biological interpretability, analytical depth, and reproducibility.

### 3.1. Overcoming the Dynamic Range of Animal and Plant Proteins

One of the most persistent challenges in proteomics is the extreme dynamic range of protein concentrations. Animal samples, such as serum or plasma, typically contain high levels of albumin, followed by immunoglobulins and other proteins. In LC-MS analysis, these abundant proteins dominate ionization and MS/MS-based acquisition, leading to the suppression of low-abundance proteins of interest. To overcome this challenge, immune-affinity depletion columns are often used to remove the top 6–14 most abundant proteins prior to digestion [53,60]. While effective, these approaches require careful validation to minimize co-depletion of protein complexes and to control batch-to-batch variability.

In plant proteomics, ribulose-1,5-bisphosphate carboxylase/oxygenase (Rubisco) dominates, accounting for nearly half of the total soluble protein in photosynthetic tissues. The abundance of Rubisco drastically limits the detection of low-copy regulatory proteins involved in signaling, stress responses, and development. Rubisco presents an analogous dynamic-range problem. PEG fractionation, protamine sulfate treatment, and affinity-based removal can reduce Rubisco abundance, but these workflows may also remove proteins that are physically associated with Rubisco complexes or stromal protein assemblies. Therefore, abundant-protein depletion should be treated as a trade-off: it can reveal low-abundance proteins, but it requires nondepleted controls, replicate quality assessment, and the transparent reporting of potential co-depletion effects [61]. Unlike plasma protein depletion, Rubisco removal must also contend with strong protein–protein interactions and the risk of losing stromal proteins that associate with Rubisco complexes. For that reason, abundant protein depletion protocols are generally used prior to these downstream processes to increase proteome depth while preserving quantitative relationships.

### 3.2. Breaking the Solvent Matrix and Extraction Barriers

Protein extraction efficiency and cleanliness are major determinants of downstream MS analysis. Animal biofluids and soft tissues generally allow for relatively easy protein solubilization using buffers supplemented with detergents, chaotropic agents, and protease inhibitors. However, lipid-rich tissues face another problem, where extracellular matrices and biofluids contain salts and endogenous proteases, requiring careful buffer optimization and rapid processing to prevent degradation [62].

On the other side, plant tissue requires harsh conditions to extract proteins due to the presence of cellulose, hemicellulose, and a lignin-made cell wall, which physically traps proteins and limits solubility. Moreover, plants produce high levels of secondary metabolites, such as phenolics, pigments, polysaccharides, and organic acids, which co-extract with proteins and interfere with enzymatic digestion, chromatographic separation, and electrospray ionization. To overcome these difficulties, plant proteomics workflows frequently use mechanical disruption (e.g., cryogenic grinding), followed by extraction with strong chaotropes and detergents or phenol-based partitioning methods. Phenol extraction efficiently separates proteins from other compounds but requires further downstream cleanup and can negatively affect labile protein recovery and PTMs [63]. These trade-offs underscore the need for extraction strategies that balance yield, purity, and biological fidelity.

### 3.3. Organelle Isolation and Membrane Protein Enrichment

Cellular proteomes are so complex, researchers often need to enrich specific cell compartments to look deeply at their protein makeup. Organelle proteomics usually performs stepwise centrifugation and then density gradients to separate structures like mitochondria, chloroplasts, nuclei, or endomembrane systems. These approaches can be used in many organisms, but differences in organelle size, density, and fragility mean the protocols must be fine-tuned for each species [64]. In plants, chloroplast isolation has to deal with high starch levels and the fact that chloroplasts are easily damaged by physical handling. In contrast, isolating mitochondria from animal cells requires very careful control of osmotic conditions to keep them intact [65]. Incomplete separation and cross-contamination remain persistent challenges, often addressed through quantitative marker analysis and computational deconvolution.

Membrane proteins are especially challenging to study because their hydrophobic transmembrane regions make them poorly soluble and they are often missed in standard proteomics workflows. To enrich and solubilize them effectively, researchers must choose detergents or surfactants that keep the proteins intact but still work well with digestion enzymes and mass spectrometry. Methods like filter-aided sample preparation, phase-transfer surfactants, and organic solvent-assisted digestion have greatly increased the number of membrane proteins that can be detected and analyzed [66]. However, variability in detergent removal efficiency and digestion completeness continues to limit reproducibility. The standardization of enrichment protocols across laboratories and kingdoms remains a critical unmet need for comparative proteomics.

## 4. Maximizing Animal Health and Productivity Through Proteomics

### 4.1. Livestock Omics for Enhancing Meat Quality, MILK Production, and Growth Efficiency

Livestock production is essential for global agriculture, as it is a significant source of milk, meat, wool, and leather. The need for animal-source foods and aquaculture products is expected to increase in many regions in the near future. This increase increases the need for production systems that improve efficiency while decreasing environmental burden. In the context of a One Health framework, maximizing livestock growth efficiency is no longer just an economic goal but a vital mechanism for reducing the environmental footprint of animal agriculture and securing global food systems amid changing climatic pressures. Proteomics can play a key role in linking molecular phenotypes with feed efficiency, fertility, disease resistance, heat tolerance, welfare, and product-quality traits. Combining omics technologies, such as transcriptomics, genomics, and proteomics, into livestock research represents a significant shift in livestock production and reproductive management [67]. However, a critical challenge remains in bridging the gap between high-throughput data and actual farm-scale phenotypic outcomes. Proteomics is one of the most cutting-edge omics technologies, enabling a detailed study of functional proteins and their diverse proteoforms to provide crucial information on livestock functional properties and the nutritional value of meat and dairy products [68].

Meat quality is a crucial factor in determining the market value of livestock. The composition of muscle tissue originally determines factors such as flavor, color, texture, and tenderness. Mass spectrometry and protein profiling permit the accurate identification of proteins and specific post-translational proteoforms involved in muscle development and of pathways that influence meat quality and energy metabolism [69,70]. Proteomic analysis of muscle tissue in livestock has successfully identified biomarkers associated with post-mortem tenderization and intramuscular fat content, directly influencing consumer satisfaction. The tenderness of meat is influenced by varying levels of glycolysis, which affect meat quality through pH and water-holding capacity [71,72]. For example, during glycolysis, the phosphorylated proteoform glycogen phosphorylase (GP) catalyzes glycogen degradation into glucose-1-phosphate. Critically, it is this specific modification, rather than total protein abundance, that accelerates glycolysis and leads to tougher meat [73].

**Figure 2:**
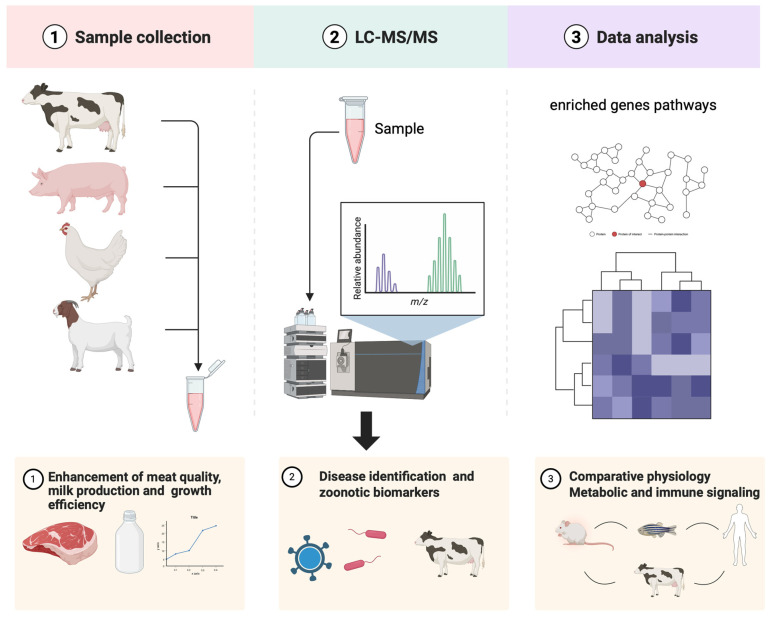
Schematic representation of the proteomics workflow in animal health and productivity. The upper panel delineates the fundamental stages of proteomic data generation and curation, including biological sampling, standardized sample preparation, mass spectrometric data acquisition, and bioinformatic storage. The lower panel illustrates the diverse translational applications of these data in livestock science, such as the enhancement of meat quality, lactogenesis, and growth efficiency, and the role of proteomics in clinical diagnostics through the identification of disease-specific signatures and zoonotic biomarkers, as well as its utility in characterizing complex signaling pathways in comparative physiology, metabolism, and immunity [67,77,78].

In the dairy sector, milk quality is a vital component of livestock productivity. The protein profile of milk is a critical indicator of its nutritional and functional value, and proteomics has the potential to elucidate the molecular factors that affect milk composition and yield. Proteomics analysis of mammary gland tissue and milk has elucidated the molecular factors behind milk composition, secretion, and lipid metabolism [74]. Proteomics also facilitates the evolution of functional milk products supplemented with bioactive peptides. Proteomic studies have identified whey proteins, caseins, and enzymes involved in lactose and fat synthesis as key parts of milk formation [75]. Understanding of these milk and meat proteoforms allows us to map how environmental stress or alternative, climate-resilient feedstocks limit animal product quality at the molecular level, bridging the gap between ecosystem health and human nutrition. The rapid advances in molecular biology techniques have significantly changed livestock breeding approaches, leading to a shift from conventional breeding to advanced genetic and molecular methods. Proteomics provides an opportunity to understand the dynamic protein abundance associated with feed modification and growth efficiency. Breeders can utilize this information to improve productivity while decreasing the environmental impact of sustainable livestock systems [76] (Figure 2).

### 4.2. Identifying Biomarkers for Zoonotic Diseases and Animal Welfare

Proteomics has emerged as a crucial tool for diagnosing and controlling diseases in livestock production. It shows promise for identifying biomarkers to diagnose and monitor diseases in domestic animals. High-resolution mass spectrometry is well-suited for biomarker discovery because it enables the large-scale analysis of proteins and their modifications in body fluids, cerebrospinal fluid, and tissues [68,77]. However, the critical challenge in veterinary diagnostics is not just identifying a protein but distinguishing between the healthy and disease-associated proteoforms (e.g., specific glycosylation or phosphorylation states) that appear during early infection. Managing this analytical gap is critical to achieving comprehensive One Health surveillance, as the early detection of proteomes in household herds serves as a frontline defense against zoonotic spillover into wildlife or human populations. These biomarkers can be useful for diagnosing disease early, predicting disease vulnerability, assessing treatment effectiveness, and assessing animal health-biomarkers identified across several health conditions, including infectious diseases and metabolic disorders [79]. Proteomics is a powerful approach for identifying biomarkers of infectious diseases in animals, such as foot-and-mouth disease, avian influenza, Mycobacterium tuberculosis, mastitis, and porcine reproductive and respiratory syndrome virus (PRRSV) [80,81].

In addition to infectious diseases, metabolic diseases such as chronic kidney disease, osteoarthritis, fatty liver disease, and ketosis are common in high-producing livestock. A proteomics study reveals that acyl-CoA synthases, a biomarker for ketosis, a metabolic disorder in cow blood and milk, are elevated when cows mobilize extra fat during early lactation [82,83]. Beyond infectious diseases and metabolic diseases, proteomics is extremely useful for evaluating animal welfare. By monitoring the specific proteoforms of acute-phase proteins and heat shock proteins, veterinarians can differentiate between acute environmental stress and chronic physiological instability, ultimately helping secure the stability of the food supply chain [77,84]. By studying welfare through these molecular perspectives, proteomics can be directly connected with macro-environmental changes. For example, severe heat waves, resource scarcity, and measurable physiological stress suggest that an accurate tool is needed for assessing and mitigating animal vulnerability within a destabilized ecosystem.

### 4.3. Using Animal Models to Understand Metabolic and Immune Signaling

Comparative physiology holds that studying diverse species can uncover fundamental biological mechanisms that are often obscured in humans. Proteomics in comparative physiology provides a foundation that enables the scientific community to move beyond static genetic blueprints to dynamic proteoform shifts, and signaling pathways across species, offering a real-time view of molecular adaptation [85]. By tracking conserved protein networks across divergent taxa, comparative proteomics serves as a modern Rosetta Stone for the One Health framework, revealing how different branches on the tree of life have evolved to solve identical environmental and pathological challenges. In metabolic signaling, proteomics tools have revolutionized the understanding of energy homeostasis. Using an animal model that shows severe phenotypes. For example, migrating birds that carry the lipid-intensive metabolism, and the dramatic shifts in insulin sensitivity seen in hibernating Ursids, reveal novel protein interactions that control energy homeostasis and cellular resilience [78,86]. A critical perspective here is that metabolic resilience in these ‘natural knockouts’ is often regulated by switching between protein variants rather than by changes in gene sequence and these findings are valuable for addressing human metabolic disorders like obesity and diabetes.

Similarly, the study of immune signaling via a comparative lens provides a broader perspective on the evolution of host defense. Proteomics enables the characterization of the “interactome,” the complex network of protein–protein interactions underlying the innate and adaptive immune responses in non-traditional models such as zebrafish, bats, and even invertebrates [87]. Critically, comparative studies of bats’ immune genes suggest that the unique proteoforms of interferon-signaling proteins allow them to tolerate high viral loads without a catastrophic proinflammatory response [88,89]. In contrast, by quantifying cytokines and chemokines, comparative proteomics identifies immune system checkpoints, and these evolutionary adaptations allow us to suggest novel ways to modulate human immune signaling in cases of autoimmune disease or infection [90,91].

Ultimately, the core analytical principles derived from these animal systems reveal that cellular stress responses operate on conserved pathways that transcend kingdom boundaries. Cross-kingdom integration within the One Health framework focuses on functional modules rather than one-to-one orthologs. Plants and animals have different receptors and tissue structures, yet both use basic mechanisms to handle stress, such as rapid post-translational regulation, redox control, proteostasis, membrane trafficking, and metabolic changes. In crops, factors such as drought, salinity, heat, and pathogen attacks impact various biological processes, including phosphorylation, antioxidant systems, transporters, and defense proteins. Similarly, in animals, issues such as infection, heat stress, metabolic strain, and poor welfare affect acute-phase proteins, heat-shock proteins, complement and coagulation factors, mitochondrial metabolism, and tissue remodeling pathways. These responses become relevant to One Health when measured across related areas, such as plant–feed–livestock systems, vector–host interfaces, rhizosphere–soil systems, aquaculture–water systems, or wildlife–livestock–human interactions. Measuring these molecular changes across different areas provides useful insights for developing health strategies that address disease risk and environmental effects.

## 5. Plant Proteomics for Crop Resilience and Food Security

Plant proteomics is a major tool for addressing two of the biggest concerns in the field of agriculture: climate change and food security in a growing world population. As mentioned, plants cannot move, but their proteomes have shown considerable flexibility. One of the major contributors to the flexibility of plant proteomes is the proteoforms of a protein, which result from the different molecular forms of a protein that come about because of genetic variation, alternative splicing, and post-translational modification (PTM). To understand complex signaling mechanisms, advanced mass spectrometry (MS) techniques, such as Data Independent Acquisition (DIA) and phosphoproteomics, are employed. However, a major problem is that total protein content does not necessarily reflect biological activity, because a plant cell’s functional status is determined by a particular proteoform of a protein, not by the total content of that protein in the cell.

### 5.1. Abiotic Stress and the Reprogramming of the Proteome

The plant response to abiotic stressors such as drought, salinity, and extreme temperatures involves a rapid reprogramming of the proteome across pre- and post-translational levels. These signals typically propagate from initial Ca^2+^/ROS and hormonal cues to deep transcriptomic reprogramming. Abiotic stress induces the expression of “resilience proteins,” including Late Embryogenesis Abundant (LEA) proteins, heat shock proteins (HSPs), and antioxidant enzymes (e.g., SOD, CAT) that moderate reactive oxygen species (ROS) damage [92,93,94,95]. Quantitative proteomics has revealed that salt tolerance is often governed by the differential abundance of ion transporters (e.g., HKT and NHX families) and the Salt Overly Sensitive (SOS) signaling pathway [96]. The SOS pathway utilizes a calcium-responsive SOS3-SOS2 protein kinase complex to control the activity of SOS1, a plasma membrane $Na^+^/H^+^ antiporter [97]. SOS3, a myristoylated calcium-binding protein, senses salt-elicited Ca^2+^ signals and activates the SOS2 serine/threonine protein kinase [98]. SOS2 subsequently phosphorylates SOS1, increasing its Na^+^/H^+^ exchange activity, which is essential for maintaining ionic homeostasis [99]. However, critically, the levels of abundance do not provide a full functional map, for example, the SOS pathway has shown that the formation of specific phospho-proteoforms is more indicative of salt tolerance than the total amount of SOS1 protein.

### 5.2. Translating the Proteomic Architecture of Plant Immunity

Proteomics has been pivotal in characterizing plant responses to biotic stress [100], particularly in defining the molecular transitions underlying the shift from PAMP-triggered immunity (PTI) to effector-triggered immunity (ETI), conceptualized in the zigzag model of plant defense [101]. The frontline defense, PTI, begins when cell-surface pattern-recognition receptors (PRRs), such as FLAGELLIN-SENSING 2 (FLS2, recognizing flagellin) and Chitin Elicitor Receptor Kinase 1 (CERK1, recognizing chitin), recruit co-receptors like BRI1-ASSOCIATED RECEPTOR KINASE 1 (BAK1) to detect conserved microbial features (PAMPs) [102]. High-resolution MS identifies pathogenesis-related (PR) proteins and maps rapid phosphorylation cascades mediated by Mitogen-Activated Protein Kinases (MAPKs) following recognition [103]. Pathogens often circumvent PTI by secreting effectors, leading to Effector-Triggered Susceptibility (ETS) [104].

**Figure 3:**
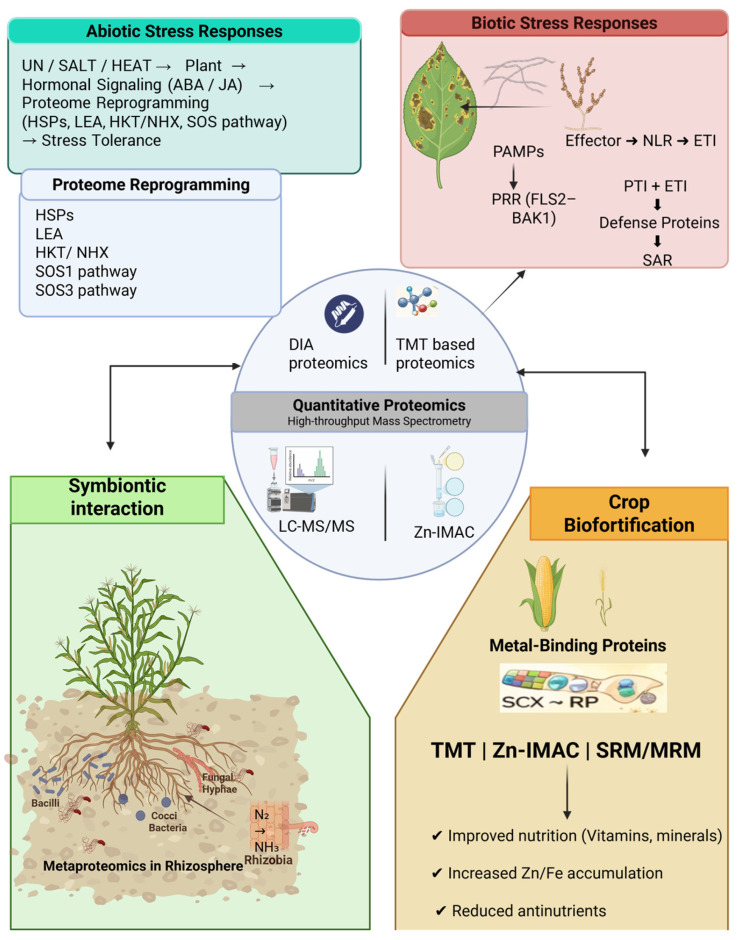
Quantitative proteomics plays a crucial role in studying plant stress responses, symbiotic interactions, and crop biofortification. The illustration depicts how plants respond to abiotic and biotic stresses, analyzed through quantitative proteomics techniques. Abiotic stress triggers ABA (abscisic acid) and JA (jasmonic acid) signaling pathways, resulting in proteome reprogramming that involves heat shock proteins (HSPs), late embryogenesis abundant proteins (LEA), ion transporters like HKT (high-affinity K^+^ transporter) and NHX (Na^+^/H^+^ exchanger), as well as the SOS (Salt Overly Sensitive; SOS1–SOS2–SOS3) pathway, all working together to sustain ionic balance and stress resilience. In response to biotic stress, plants recognize pathogen-associated molecular patterns (PAMPs) via pattern recognition receptors (PRRs, such as the FLS2–BAK1 complex), initiating PAMP-triggered immunity (PTI). Pathogen effectors are detected by intracellular NLRs (nucleotide-binding leucine-rich repeat proteins), which activate effector-triggered immunity (ETI) and subsequent defense mechanisms like pathogenesis-related proteins (PR), reactive oxygen species (ROS), vesicle secretion, and systemic acquired resistance (SAR). Symbiotic interactions in the rhizosphere include nitrogen fixation by rhizobia (N_2_ → NH_3_) and microbial community analysis through metaproteomics. Crop biofortification strategies focus on metal-binding and transport proteins to boost micronutrient levels (such as Zn and Fe) and reduce antinutrients. levels (such as Zn and Fe) and reduce antinutrients. Proteomics workflows encompass DIA (data-independent acquisition), TMT labeling, LC-MS/MS (liquid chromatography–tandem mass spectrometry), Zn-IMAC (zinc immobilized metal affinity chromatography), SCX (strong cation exchange), RP (reverse phase chromatography), and targeted SRM/MRM (selected/multiple reaction monitoring) for precise protein quantification [22,92,100,113].

Plants can then recognize these effectors through intracellular nucleotide-binding leucine-rich repeat (NLR) receptors [105]. This triggers a highly specific and potent ETI response. This response is usually accompanied by an areal hypersensitive response (HR) and Systemic Acquired Resistance (SAR), which sensitizes the tissues to future infections. Proteomics on the apoplastic proteome has shown key defense responses. For example, the plant sends out PR-3 (chitinase) and PR-5 (thaumatin/osmotin), which burst through the membranes of the fungus and stop the growth of the hyphal cells. Furthermore, when PTI is activated in the plant cell, there is a shedding of pattern recognition receptors in the extracellular domain and an increase in exosome secretion into the apoplastic washing fluid (AWF). These defense responses occur in the extracellular compartment and play a role in controlling the proliferation of the pathogen [106] (Figure 3).

### 5.3. Quantifying the Blueprints of Crop Biofortification

Quantitative mass spectrometry-based proteomics plays a central role in crop biofortification. This technique is used to enhance the nutritional value of food crops and alleviate micronutrient deficiencies. This is done through precise molecular techniques that monitor the accumulation of key minerals and vitamins in the seed proteome. For example, through the study of the proteome of cereal grains, scientists were able to identify key metal–protein interactions that store iron and zinc in the endosperm [107].

Using advanced methods like zinc-affinity immobilized metal affinity chromatography (Zn-IMAC), researchers have shown that storage proteins such as B-hordeins and 7S globulins act as the main reservoirs for zinc inside grains. Beyond just storing minerals, proteomics plays a key role in boosting protein quality [108]. By fine-tuning the balance of essential amino acids like lysine and methionine, which are often scarce in maize and legumes, scientists can improve nutritional profiles [109,110]. Yet, analyzing seed proteomes is challenging. The sheer complexity and wide concentration range make it tough, especially since storage proteins dominate in the 10–130 kDa range [111].

These abundant proteins may obscure the presence of low-copy regulatory, signaling, and metabolic proteins; hence, proteome coverage is restricted. For improved analytical depth, multidimensional separations are performed using a combination of orthogonal chromatographic strategies, which include the use of a Strong Cation Exchange (SCX) followed by a Reversed Phase (RP) chromatography approach prior to the use of tandem mass spectrometry [112]. This 2D approach improves the resolution of peptides and enhances the overall efficiency of the separation. Using the increased resolution power provided by the 2D approach, it is much easier to identify the low-abundance proteins—the key proteins involved in seed metabolism and development. Quantitative MS strategies such as tandem mass tag (TMT) labeling are employed to monitor the levels of seed storage proteins (SSPs) and pinpoint the key targets for metabolic engineering [113]. TMT labeling can handle up to 11 samples at once. Isobaric tags react with primary amines in peptide digests, so when you run the MS1 spectrum, signals from each peptide add up together [113]. Upon fragmentation, condition-specific reporter ions with different *m*/*z* values are generated, enabling a precise relative quantification with minimal missing data. Proteomic tools monitor and reduce the presence of anti-nutritional factors and allergens in crops [114]. Targeted workflows, such as Selected Reaction Monitoring (SRM/MRM), provide high-sensitivity detection of specific allergenic isoforms, such as gluten from barley or wheat, ensuring that biofortified products are both nutritious and safe for consumption [115].

### 5.4. Proteomic Exchange in Plant-Microbe Symbiosis

Symbiotic plant–microbe interactions represent highly coordinated biological systems that optimize nutrient acquisition and maintain agroecosystem productivity [116]. The molecular and functional structure of plant and microbe interactions, especially with legumes and rhizobia that have a localized biological nitrogen fixation site (root nodule) has been investigated through proteomic analysis. The double-proteomic approach using high-resolution LC–MS/MS enables measurement of both plant and bacterial proteins within the same compartment and enables the temporal tracking of infection, differentiation between the two organisms, and metabolic interactions [117,118]. A major bioinformatic problem that is encountered in these studies is the ortholog problem, which refers to the difficulty that is encountered due to the conserved sequences between the plant and the endosymbionts. The first stages of the symbiosis are marked by the induction of rhizobial Nod factors via flavonoids and a rapid restructuring of the host root hair proteome by cytoskeletal regulators, vesicular trafficking elements, and early nodulins related to organogenesis [119,120]. Following endocytosis, bacteroids are enclosed within the symbiosome membrane, whose specialized proteome includes aquaglyceroporins, dicarboxylate transporters, ammonium channels, metal ion transport systems, and H^+^-ATPases that collectively regulate nutrient flux and electrochemical gradients [121]. The microaerobic environment required for nitrogenase activity is maintained by high concentrations of leghemoglobin, which ensures there is sufficient oxygen for oxidative phosphorylation while preventing enzyme inactivation. Metabolic coupling is sustained by plant-derived C_4_-dicarboxylates that support bacteroid respiration and ATP generation, with fixed ammonia assimilated via the glutamine synthetase/glutamate synthase cycle and transported systemically as amides or ureides. Parallel proteomic analyses of arbuscular mycorrhizal symbiosis reveal extensive remodeling of the periarbuscular membrane, including mycorrhiza-inducible phosphate transporters and proton pumps that facilitate high-affinity phosphorus uptake through the symbiotic pathway [120,122]. At the rhizosphere level, both secretome and metaproteomic profiling indicate that plants actively recruit PGPR (plant growth-promoting rhizobacteria) through specific exudation patterns that facilitate nutrient mobilization, phytohormonal regulation, and the induction of systemic resistance. These two distinct forms of proteomics provide a molecular description of the mechanisms that mediate symbiotic efficiency, enabling the engineering of nutrient-responsive crops [123] and biologically optimized fertilizer practices (Figure 3.).

Microbiome and environmental proteomics are vital for a One Health approach because microbial communities connect host health, agricultural output, antimicrobial resistance, and ecosystem functions. Metaproteomics directly measures microbial proteins expressed, allowing for differentiation between a community’s functional state and its taxonomic potential. In soil and rhizosphere environments, it can detect enzymes involved in nitrogen cycling, phosphorus mobilization, carbon processing, and plant growth promotion. In animal and human interactions, gut, respiratory, milk, wastewater, and farm-environment metaproteomes can identify microbial effectors, virulence factors, antimicrobial resistance proteins, and host response proteins. In aquatic environments, water and fish/shellfish proteomes can indicate hypoxia, pollutants, pathogen presence, and welfare status. These uses extend the scope from separate plant and animal proteomics to ecosystem-level molecular monitoring. Insects serve as pollinators, pests, disease vectors, decomposers, and increasingly as alternative protein sources for feed and food. MS-based insect proteomics can detect detox enzymes, immune proteins, salivary or venom components, reproductive factors, and pathogen-interaction pathways linked to vector capacity and pest control. Aquaculture proteomics similarly connects food security with animal welfare and environmental health by identifying proteins related to growth, smoltification, immune response, heat stress, hypoxia, nutrition, and product quality in fish and shellfish. Including these areas broadens the framework beyond terrestrial livestock and crops, strengthening the One Health perspective.

## 6. Proteogenomics and Artificial Intelligence

### 6.1. Proteogenomics for Refining Genome Assembly Using MS-Derived Peptide Evidence

Proteogenomics has become an important complementary strategy for refining genome annotation because MS-derived peptide evidence can validate predicted protein-coding genes, support exon boundaries, identify translated small open reading frames, and reveal sample-specific or non-canonical translation events. However, it should not be described as replacing genome annotation pipelines or as the primary approach to genome assembly. Its strongest role is as an evidence layer that integrates with long-read DNA sequencing, RNA sequencing, comparative genomics, and curated annotation. By 2026, proteogenomics emerged as the primary approach for refining genome assemblies [124]. Using peptide evidence from mass spectrometry, researchers can predict and validate Open Reading Frames (ORFs) and identify non-canonical translation products, such as those resulting from alternative splicing or stop-codon read-through, that conventional genomic methods might miss [125].

In cancer research, proteogenomics has been instrumental in investigating the dark proteome, and sample-specific pipelines can reveal thousands of novel peptides, many of which serve as neoantigens for personalized vaccine development [126,127]. In plant science, where polyploidy (such as in wheat) and repetitive sequences complicate genome assembly, proteogenomics approaches are essential for correcting misannotated gene models. Combining long-read DNA and RNA sequencing with multi-protease mass spectrometry data ensures that observed protein data align with genome predictions and can capture all protein-coding potential across various species [128,129]. However, although proteogenomics improves genome annotation and helps discover new coding sequences, it is not necessarily capable of solving problems related to proteoform diversity. The merging of information from genomics and peptides is primarily conducted at the level of sequence information, neglecting the intricacies associated with post-translational modification and protein isoforms. Therefore, proteogenomics alone is insufficient for comprehensive proteome function characterization.

### 6.2. Artificial Intelligence and Deep Learning for Predicting Structure and Function

The emergence of AI-driven tools, such as AlphaFold, has permanently altered the landscape of structural biology from a static structure to a detailed understanding of protein dynamics. AlphaFold 3 extends structure prediction from single proteins toward the joint modeling of biomolecular complexes that can include proteins, nucleic acids, small molecules, ions, and modified residues. This is highly relevant to proteomics because structural models can help interpret peptide evidence, variants, ligand binding, and the potential effects of selected post-translational modifications. Nevertheless, these predictions should be treated as hypotheses requiring experimental validation. Current structure-prediction tools do not fully capture proteoform-specific dynamics, intrinsically disordered regions, transient interactions, local pH, crowding, or tissue-specific biochemical environments. For One Health proteomics, the priority is therefore to integrate AI-based structural hypotheses with experimentally measured proteoforms, PTMs, and interaction data [130]. However, a crucial analysis of these tools indicates a persistent gap. While new AI tools can predict the most stable folded state of a protein, They often struggle to account for the structural fluidity of inherently disordered regions (IDRs) or the temporary conformational changes induced by the cellular environment conditions.

We are now predicting the effects of post-translational modifications (PTMs) and point mutations on protein stability with unprecedented accuracy. In human medicine, AI-based structural predictions and tools such as DeepMVP and PTMGPT2 accelerate drug discovery by revealing cryptic pockets in previously considered undruggable proteins. In agriculture and plant sciences, generative AI is used to design new proteins with improved thermal stability or specific catalytic properties, such as drought-resistant enzymes in crops [131]. Deep learning frameworks such as DeepLC have become essential to the proteomic workflow, enabling the silico prediction of peptide retention times. This digital twin of the mass spectrometer improves the sensitivity of DIA experiments, connecting sequence information to biological function phenotypes [132].

Looking forward, one of the primary goals for the next generation of One Health proteomics must be to introduce the live cell context into AI models. Current structural prediction tools are mostly independent of local biochemical conditions, such as pH changes in plant vacuoles or crowding effects in animal mitochondria and other organelles. To advance the field, we need to shift from predicting isolated protein structures to modeling proteoform-specific interactomes. This means using AI to predict how a single phosphorylation or glycosylation event can alter an entire signaling network. By integrating static structural protein data with dynamic mass spectrometry, a wide profiling of post-translational modifications (PTMs) can be obtained, and AI can evolve from a predictive tool to a functional engine for engineering biological strength across different taxa.

### 6.3. Ethical Implications, Privacy, Biosecurity and Digital Equity

Reproducible One Health proteomics requires standardization at both the laboratory and data levels. Minimum reporting should include sample origin, collection time, environmental context, extraction protocol, digestion conditions, instrument method, acquisition mode, search database, false-discovery-rate strategy, missing-data handling, normalization method, and quality-control metrics. Data deposition should use community repositories and open standards wherever possible, including ProteomeXchange resources, PRIDE, MassIVE, PeptideAtlas, jPOST, iProX, and Panorama Public, together with formats such as mzML, mzIdentML, mzTab, and SDRF-Proteomics for experimental metadata. These practices make datasets FAIR and are particularly important for cross-kingdom comparisons, where sample metadata and database construction strongly influence protein inference. The adoption of FAIR data principles also enables meta-analyses across studies, which are essential for identifying conserved proteomic signatures of stress, disease, and resilience across the tree of life (Table 1).

Use of AI in proteomics raises significant ethical concerns. In human proteomics, going from static genomics to dynamic, context-specific proteomics raises concerns about privacy-related functions. AI models can now predict a person’s current health or lifestyle from a single blood sample, raising the risk of proteomic fingerprinting that insurance companies might misuse, while AI tools can assist in designing new vaccine candidates, they could also be used to create new toxins. To mitigate this, high-level protein design software should include digital watermarking in synthetic sequences to enable traceability. Lastly, we must contain Digital Colonialism, where foreign AI exploits the biological resources of the Global South. Promoting open-source proteomics and building local computing centers are essential to making the proteomic revolution an inclusive global movement.

## 7. Future Perspective and Improvement

Integrated proteomics combines techniques such as LC/MS, 4D proteomics, and AlphaFold, which produce extensive, complex data. To fully understand this data, future advances will focus on developing robust, scalable data analysis pipelines and specialized databases capable of managing large, intricate datasets. These systems will improve data processing, integration, annotation, and visualization. As these tools develop, they will support more complex queries and provide deeper biological insights, ultimately boosting hypothesis generation and discovery. A major future direction will be to move from static descriptions of proteins to a dynamic understanding of their behavior. Combining computational methods, such as molecular dynamics simulations, with experimental techniques like LC/MS and AI-driven structure prediction will allow researchers to observe conformational flexibility, transient interactions, and the functional consequences of PTMs in physiologically relevant environments.

From a translational perspective, the widespread adoption of proteomics platforms is anticipated to significantly impact clinical research and practice. The ability to identify and quantify disease-specific signatures will improve biomarker discovery and enable more precise disease identification. In turn, this will support the development of personalized therapeutic strategies tailored to individual proteomic profiles. However, implementation will need to address challenges related to high instrumentation costs, computational demands, data interpretation, and interdisciplinary collaboration. The major areas for implementation will include extending the analytical capacity of 4D proteomics to larger, more complex proteomic systems, improving structural dynamics within predictive models, and establishing high-quality, interoperable databases. The development of hybrid computational and experimental frameworks and standardized analytical workflows will further enhance reproducibility and accessibility across laboratories.

Proteomics will continue to provide unique, functionally relevant insights by directly analyzing protein diversity, functional variation, and molecular interactions. The integration of proteomics with emerging fields such as single-cell and spatial omics will enable the resolution of protein heterogeneity at an extraordinary level of detail, providing new insights into cellular and tissue-level complexity. Looking ahead, advances in real-time MS technologies, AI-driven modeling, and high-performance computing will likely position proteomics as a crucial element of systems biology. In the near future, proteomics-specific biomarkers and therapeutic targets could become standard components of precision medicine, especially for complex diseases such as cancer and neurodegeneration, as well as for plant-pathogen-specific biomarkers. Although technical and translational challenges remain, continuous innovation and cross-disciplinary collaboration will push the field toward more accurate, scalable, and clinically valuable applications, ultimately unlocking the full potential of proteomics in next-generation biomedical research and personalized healthcare.

## Figures and Tables

**Table 1:** Table summarizes selected computational tools and platforms used in modern proteomics, including function and application in proteomics research.

Tool/Platform	Primary Function	Proteomics Application	References
AlphaFold 3	Joint biomolecular complex structure prediction	Structural interpretation of peptide evidence, PTM effects, ligand binding	[24]
DIA-NN	Neural network-based DIA data analysis	Deep proteome coverage, spectral deconvolution, cross-kingdom datasets	[32]
DeepLC	Peptide retention time prediction	In silico spectral library building for DIA, improves identification sensitivity	[132]
DeepMVP	Cryptic pocket identification in protein structures	Drug target discovery; identification of undruggable protein regions	[25]
MaxQuant/PASEF	Ion mobility-enhanced peptide quantification	4D proteomics in complex biological samples across kingdoms	[133]

## Data Availability

No new data were created or analyzed in this study.

## Abbreviations

The following abbreviations are used in this manuscript:

MS	Mass spectrometry
DIA	Data-independent acquisition
DDA	Data-dependent acquisition
PTM	Post-translational modifications
IMS	Ion mobility separation
M/Z	Mass-to-charge ratio
MALDI	Matrix-assisted laser desorption/ionization
LCM	Laser capture microdissection
GP	Glycogen phosphorylase
SOS	Salt Overly Sensitive
PTI	PAMP-triggered immunity
ETI	Effector-triggered immunity
ETS	Effector-triggered susceptibility
SCX	Strong Cation Exchange
PGPR	Plant growth-promoting rhizobacteria
